# Leveraging Energy Harvesting and Wake-Up Receivers for Long-Term Wireless Sensor Networks

**DOI:** 10.3390/s18051578

**Published:** 2018-05-15

**Authors:** Fayçal Ait Aoudia, Matthieu Gautier, Michele Magno, Olivier Berder, Luca Benini

**Affiliations:** 1 University of Rennes, CNRS, IRISA, 6 rue Kerampont, F-22305 Lannion CEDEX, France; faycal.ait-aoudia@irisa.fr (F.A.A.); olivier.berder@irisa.fr (O.B.); 2 ETH Zurich, Integrated Systems Laboratory, Gloriastrasse 35, 8092 Zurich, Switzerland; michele.magno@iis.ee.ethz.ch (M.M.); lbenini@iis.ee.ethz.ch (L.B.)

**Keywords:** wireless sensor networks, energy harvesting, wake-up radio, MAC protocols, energy management

## Abstract

Wireless sensor nodes are traditionally powered by individual batteries, and a significant effort has been devoted to maximizing the lifetime of these devices. However, as the batteries can only store a finite amount of energy, the network is still doomed to die, and changing the batteries is not always possible. A promising solution is to enable each node to harvest energy directly in its environment, using individual energy harvesters. Moreover, novel ultra-low power wake-up receivers, which allow continuous listening of the channel with negligible power consumption, are emerging. These devices enable asynchronous communication, further reducing the power consumption related to communication, which is typically one the most energy-consuming tasks in wireless sensor networks. Energy harvesting and wake-up receivers can be combined to significantly increase the energy efficiency of sensor networks. In this paper, we propose an energy manager for energy harvesting wireless sensor nodes and an asynchronous medium access control protocol, which exploits ultra-low power wake-up receivers. The two components are designed to work together and especially to fit the stringent constraints of wireless sensor nodes. The proposed approach has been implemented on a real hardware platform and tested in the field. Experimental results demonstrate the benefits of the proposed approach in terms of energy efficiency, power consumption and throughput, which can be up to more than two-times higher compared to traditional schemes.

## 1. Introduction

Wireless Sensors Networks (WSNs) are today a mature technology enabling a large variety of cyber-physical system applications in environmental monitoring, healthcare, security and industrial domains. They are composed of multiple wireless sensor nodes that monitor an environment and wirelessly send information data to one or more remote hosts called sinks. A wireless sensor node is made of several components: a processing unit, memory, sensors, a transceiver and an energy source [1]. Usually, these devices are battery-powered and therefore have a limited lifetime, making energy one of the most precious resources, especially in scenarios where the network is expected to work for several months or even years.

To tackle this problem, a successful approach is Energy Harvesting (EH), which allows the nodes to be powered by environmental energy sources such as sunlight, wind, vibration, water flow, etc. Using EH, it is possible to increase the WSN lifetime by an order of magnitude with respect to traditional battery-powered approaches and to achieve the Energy Neutral Operation (ENO) state, i.e., the amount of harvested energy is greater than or equal to the amount of consumed energy over long periods of time [2,3,4,5]. Obviously, adequate energy management techniques remain a key feature for enabling EH-WSNs.

Harvested energy is by nature unpredictable and intermittent [5], making energy management strategies crucial in EH-WSNs. Efficient energy management requires both careful hardware design (by selecting optimal circuits) and energy-aware algorithms (e.g., network protocols). As wireless communication is usually the primary consumer of energy in WSNs [6], many energy management techniques focus on improving the efficiency of radio communication. One of the most widespread techniques is duty-cycling, in which the transceiver is periodically turned on and off according to a schedule [7], and the proportion of time during which the node is active is termed the duty-cycle. If duty-cycling considerably increases the lifetime of WSNs, it does not cancel the two primary sources for unnecessary energy consumption in WSNs: overhearing, i.e., reception of frames intended for other nodes, and idle listening, i.e., time during which the transceiver is active, but not receiving nor transmitting. A widespread solution to reduce these energy wastes is the design of adaptive communication schemes that modify their duty-cycling according to the available energy [8,9,10]. In this work, the mainstream contribution is to annul these wastes by associating EH-WSN and the Ultra Low Power (ULP) Wake-up Receiver (WuRx).

Recent circuit-level advances have made possible the realization of ULP WuRx circuits that can efficiently “wake up” a node when a specific signal, called the Wake-up Beacon (WuB), is sent by a neighboring node [11,12,13,14,15,16]. Moreover, many ULP WuRx embed address matching features, which allow nodes to wake up only a specific node and not all their neighbors [17,18]. The main feature of ULP WuRx is the continuous listening of the wireless medium while keeping the main transceiver in the sleep state. When the node is in sleep state, the ULP WuRx is the only active component listening for WuBs and consuming power in the micro- or nano-Watt range, which is negligible when compared with the main radio that consumes milliwatts when active [11,12,13,17,18]. ULP WuRx were already successfully used in the context of wireless body area networks [19,20] in which the limitations in terms of power budget are stringent [10].

Combining ULP WuRx with recent improvements in EH allows a significant increase of network lifetime [21]. However, in order to efficiently take advantage of this combination, this paper proposes to jointly design dedicated energy management strategies and ad hoc communication protocols. Indeed, communication protocols need to be optimized to exploit the new potentiality offered by ULP WuRx and to deal with the intermittent harvested energy, especially Medium Access Control (MAC) protocols, whose tasks are to provide mechanisms to allow several wireless nodes to share the wireless channel medium and access the network. A global energy management system, which deals with the hardware, software and network, is needed to maximize the efficiency and to achieve the goals of long lifetime and energy neutrality.

In this paper, a novel Energy Management (EM) scheme, which optimizes the energy use in EH-WSN, is proposed in combination with a novel MAC protocol, which exploits the benefits of ULP WuRx. The proposed MAC protocol focuses on the star network topology, in which a central sink to which all the other nodes are connected collects the data [8]. It is therefore assumed that no communication from the sink to the nodes is required, which is the case in many monitoring applications [22]. The main contributions of this paper are as follows:

- A novel EM for EH-WSNs is proposed. Unlike most state-of-the-art EMs, the energy management strategy proposed in this work only requires the residual energy as an input, making it practically easy to implement on real hardware platforms.
- A novel MAC protocol, called SNW-MAC (Star Network WuRx-MAC) leveraging ULP WuRx for data-gathering star networks is proposed. SNW-MAC enables asynchronous communications, minimizes the cost of packet transmissions and allows error corrections. SNW-MAC significantly reduces the energy cost variability of packet transmissions, allowing accurate control of the consumed energy by the EM. Moreover, an analytical study of SNW-MAC scalability is presented.
- SNW-MAC and the proposed energy management scheme were implemented and evaluated in the field, using a state-of-the-art ULP WuRx [13]. The proposed scheme was evaluated in the context of indoor light energy harvesting through exhaustive experimentation.
- In order to achieve a fair evaluation of the proposed scheme, two state-of-the-art MAC protocols were also implemented on the same hardware and application scenario. Results show that ULP WuRx allow improved communication efficiency, which is exploited by the EM to achieve a higher throughput (up to more than double) compared with state-of-the-art schemes. To rigorously measure this improved energy efficiency in the context of data gathering WSNs, the Energy Utilization Coefficient (EUC) is defined and used as an evaluation metric.

The remainder of this paper is organized as follows. Section 2 describes the related work. Section 3 presents the energy management scheme and the EUC metric. Section 4 details the design of SNW-MAC and presents an analytical study of its scalability. Section 5 exposes the experimental setup used to evaluate our approach, and Section 6 presents the experimental results. Finally, Section 7 concludes this paper.

## 2. Related Work

To the best of our knowledge, no previous works have proposed a joint focus on both EM and communication with ULP WuRx. However, many papers deal with either one or the other topic. Hence, this section is split into two subsections on the two topics. The related work regarding energy management for EH-WSNs is first given, followed by a presentation of state-of-the-art MAC protocols leveraging ULP WuRx.

### 2.1. Energy Management for EH-WSNs

Research on energy management for EH-WSNs has been very prolific in recent years, and many solutions have been proposed [2,23,24,25,26,27,28,29,30,31]. They can be classified based on their requirement of predicted information about the amount of energy that can be harvested in the future, i.e., prediction-based and model-free. Prediction-based schemes require that an energy predictor [32] supplies the EM with predictions of the future harvested energy. The first EM using the prediction-based approach was introduced in 2007 by Kansal et al. [2]. In this scheme, an exponentially-weighted moving average filter is used to predict the future amount of harvested energy, and the duty-cycle is computed according to the difference between predicted and observed energy inputs. Castagnetti et al. introduced the Closed-Loop Power Manager (CL-PM) in [24], which uses two distinct energy management strategies, one for periods during which environmental energy is available and one for periods during which the harvested energy is below a fixed threshold, referred to as zero energy interval. Le et al. proposed Wake-up Variation Reduction PM (WVR-PM) [25], a variation of CL-PM that allows a node to store more energy when environmental energy is available to achieve a similar quality of service during zero energy interval periods than when environmental energy is available. Renner et al. proposed a prediction-based algorithm [33] for energy harvesting sensor nodes using a supercapacitor as the energy storage device. The algorithm consists of a prediction block, which provides the forecast of the supercapacitor energy reserve for a given consumption and a given harvest forecast. The second block implements an energy policy, which defines predicates to enforce the properties of the operation style. The algorithm was implemented on a testbed of twelve energy harvesting wireless sensor nodes organized in a multihop topology, and the field test lasted four weeks. In a previous work [26], we introduced GRAPMAN, an EM for EH-WSNs powered by pseudo-periodic energy sources that aims to achieve high average throughput while maintaining consistent quality of service, i.e., with low fluctuations with respect to time.

As the amount of energy that a sensor can harvest shows large fluctuations and is hard to predict, energy predictors suffer from significant errors, incurring overuse or underuse of the harvested energy [29]. Unlike prediction-based approaches, model-free schemes do not require any prediction or model of the energy source. LQ-Tracker [27] uses Linear-Quadratic Tracking, a technique from adaptive control theory, to adapt the duty-cycle considering only the state of charge of the energy storage device. Similarly, Le et al. proposed using a Proportional Integral Derivative (PID) controller [28]. With P-FREEN [29], Peng et al. designed an EM that maximizes the duty-cycle of a sensor node in the presence of battery storage inefficiencies. The authors formulated the average duty-cycle maximization problem as a non-linear programming problem and proposed a set of budget-assigning principles that maximized the duty-cycle by using the currently-observed energy harvesting rate and the residual energy. In a previous work [30], we proposed an approach that relies on fuzzy control theory to dynamically set the node’s consumed energy. Fuzzy rules are used to make a decision about the allocated energy considering the current state of charge of the energy storage device and the amount of harvested energy.

Yang et al. proposed AutoSP-WSN [4], a framework for Solar-Powered WSNs incorporating both energy management algorithms and communication protocols and that was evaluated in the field. However, this work differs from ours in two major ways. First, the authors introduce routing and link rate control protocols, while we focus on MAC protocols leveraging emerging ULP WuRx. Moreover, AutoSP-WSN features a prediction-based EM, while a model-free EM is proposed in this work, which suits the stringent hardware constraints of WSN devices.

Most of the previously-proposed EM have been evaluated only through simulations, without any implementations on real sensor node hardware. Therefore, many practical problems are considered out of the scope of these studies. For example, accurate energy spending mechanisms and detailed harvested energy tracking are difficult to implement, and their implementation incurs significant overhead [34]; however, many theoretical energy-harvesting adaptive algorithms assume the availability of these values. In this study, the proposed EM has been experimentally evaluated using real hardware platforms. To this aim, the proposed EM only needs the current residual energy, and it can be used with various MAC protocols.

### 2.2. MAC Protocols Leveraging Wake-Up Receivers

There has been a tremendous amount of research on the design and implementation of MAC protocols in WSNs [35]. WSN MACs can be classified into three paradigms: synchronous, pseudo-asynchronous and asynchronous. In the first approach, neighboring nodes are synchronized to wake up at the same time. However, in the context of EH-WSNs, environmental power sources provide energy that continuously varies over time and space, making synchronous approaches not optimal for such application scenarios [36]. Indeed, nodes powered by energy harvesting must be able to dynamically adapt their duty-cycle, and pseudo-asynchronous and asynchronous schemes allow each node to choose its active schedule independently of other nodes. Traditionally, pseudo-asynchronous schemes rely on duty-cycling [7], in which nodes are periodically powered on and off according to their own specific schedule while establishing on demand rendezvous using a beaconing approach.

Pseudo-asynchronous schemes can be categorized as transmitter-initiated or receiver-initiated depending upon who initiates the rendezvous [37]. In the transmitter-initiated scheme, the receiving node periodically wakes up to monitor the channel and goes back to sleep after a short wake-up duration if the channel is found to be clear. When a node has a packet to send, it transmits request-to-send signals to the destination node, each followed by a listening period. The destination node, upon waking up according to its regular schedule acquires the transmit request and answers the transmitting node by a clear-to-send message. After this rendezvous process, the data packet is sent. In the receiver-initiated scheme, the receiving node periodically wakes up and send a clear-to-send beacon. It then monitors the channel for a short duration and goes back to sleep if no signal is detected. If a node needs to transmit data, it listens to the channel for the clear-to-send beacon from the receiver and, upon reception, starts sending its data packet. Many variations of these two approaches can be found in the literature [35], and because of the underlying wake-up scheme and rendezvous process, these approaches are referred to as pseudo-asynchronous.

Fully-asynchronous communications can be achieved with the use of ULP WuRx. ULP WuRx enables the cancellation of the energy waste due to the rendezvous process and the periodic wake-ups [38] As ULP WuRx technology is still under development and relatively new, only a few research studies were conducted on designing communication protocols leveraging ULP WuRx. WUR-MAC [39] was the first MAC protocol that took advantage of ULP WuRx. The ULP WuRx and the main transceiver use separate channels, and a request-to-send/clear-to-send handshake with channel assignment is done using the ULP WuRx. By receiving each incoming request-to-send and clear-to-send frame, a node has therefore the information about which channel is used by its neighbors. When it wants to communicate, it randomly chooses a free channel and sends a request-to-send frame containing the chosen channel. As our approach does not require request-to-send/clear-to-send handshake or a similar mechanism, packet transmissions are energetically less expensive than with WUR-MAC. Zippy [18] is a flooding protocol that leverages WuRx. This protocol has been experimentally validated on real sensor nodes. However, in the context of data-gathering star networks, the flooding approach, in which each packet is sent to all neighboring nodes, is not well-suited as only the sink needs to receive the data sent by the nodes. In a previous work, we introduced OPWUM [40], an opportunistic forwarding MAC using timer-based contention for next hop relay selection and leveraging ULP WuRx. This MAC is nonetheless specifically designed for multi-hop networks, and is therefore not appropriated to star networks. In this paper, we propose a protocol dedicated to star networks, and unlike most of the previously-cited protocols, this protocol was implemented on sensor nodes and evaluated in the field.

Regarding EH-WSNs, Le et al. [38] compared the energy consumption of the TICERprotocol [37] with and without ULP WuRx. Through simulations, showed that using a ULP WuRx drastically reduces the energy cost of communications. The energy thus saved is used to increase the node throughput. This approach is nonetheless still energetically expensive, as it requires the nodes to send WuBs, which is done at high transmission power and a low bitrate because of the current WuRx sensitivity. Magno et al. proposed a power unit for WSN nodes [21], which features multi-source energy harvesting, multi-storage adaptive recharging, as well as wake-up capabilities using ULP WuRx, which enables the control and configuration of the power unit in an energy-efficient way. In this paper, the proposed ULP WuRx-based communication scheme is fully coordinated by the sink of a star network. The risk of collisions is canceled, and the energy cost of a transmission is therefore reduced and constant. This allows an accurate control of the consumed energy by the EM.

## 3. Energy Management for an Energy-Harvesting Sensor Node

In this section, we introduce a new EM for EH-WSNs, whose task is to dynamically adjust the performance of the node, evaluated in this work by the throughput, according to the current residual energy. The proposed EM can be used in collaboration with various MAC protocols. Later, we show the benefits of combining this novel EM with the SNW-MAC protocol leveraging WuRx proposed in Section 4.

**Figure 1:**
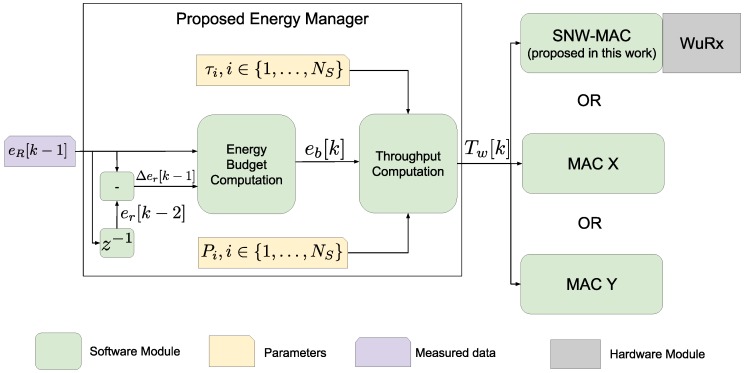
Software architecture with a detailed view of the proposed Energy Management (EM) structure. The design of the proposed Star Network Wake-up Receiver (WuRx) (SNW)-MAC protocol is detailed Section 4.

We assume that the time is divided into time slots of equal duration *T*, and the EM is executed at the end of each slot to set the throughput of the node for the next slot *k*. At each execution, the EM measures the current residual energy denoted by $e_{R}$ and sets the frequency at which the node performs sensing and sends the so-obtained data. The EM sets this frequency by adjusting the wake-up interval for the next slot denoted by $T_{WI}$, i.e., the time between two consecutive sense-and-send operations. Two submodules compose the proposed EM as shown in Figure 1. The Energy Budget Computation (EBC) module evaluates the energy that the node can consume in the next time slot *k* to remain sustainable. This amount of energy is called the energy budget and is denoted by $e_{B}\left[k\right]$. The inputs to the EBC are the residual energy at the end of the slot k−1 and the variation of residual energy, respectively denoted by $e_{R}\left[k-1\right]$ and $\Delta e_{R}\left[k-1\right]$, defined by:

(1) $$\Delta e_{R}\left[k-1\right]=e_{R}\left[k-1\right]-e_{R}\left[k-2\right].$$

The second module is the Throughput Computation (TC) module, which calculates the wake-up interval $T_{WI}\left[k\right]$ according to the energy budget $e_{B}\left[k\right]$. When the topology is a star network, the only task of a node is to perform a measurement and to send the so-generated data to the sink. In multi-hop networks, each node must also relay packets sent by other nodes. Only the TC is specific to star network applications, and the EM can therefore easily be adapted to multi-hop scenarios by designing a module replacing the TC that allocates the energy budget among the sensing and the relaying tasks, required by multihop networks.

### 3.1. EBC Design

**Figure 2:**
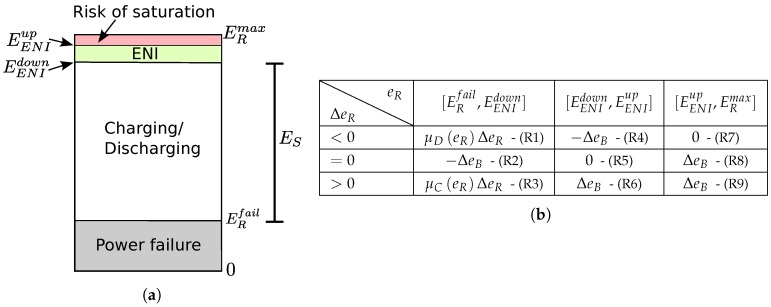
Energy storage levels and rule base used to compute $\delta e_{B}$. ENI, Energy Neutral Interval. (**a**) Energy storage device levels. (**b**) Rule base used to compute $\delta e_{B}$.

Most of the EMs presented in the literature assume the availability of the harvested and consumed energy values [2,24,25,30]. However, precise tracking of these values is difficult, and their implementation incurs high overhead [34]. Therefore, an EM that only requires the current amount of residual energy is proposed in this work. The aim of the EBC is to keep the device in the ENO-MAX state, i.e., the amount of consumed energy equals the amount of harvested energy over a long period of time [27], by dynamically adapting the energy budget. Four residual energy levels of the energy storage device are defined and shown in Figure 2a. $E_{R}^{max}$ is the energy storage capacity, and $E_{R}^{fail}$ is the hardware failure threshold, which identify the minimal energy level ensuring the correct supply of the device. $E_{R}^{fail}$ and $E_{R}^{max}$ are hardware dependent. An Energy Neutral Interval (ENI) is defined by two energy thresholds $E_{ENI}^{up}$ and $E_{ENI}^{down}$ such that $E_{R}^{fail}<E_{ENI}^{down}<E_{ENI}^{up}<E_{R}^{max}$, as illustrated by Figure 2a. If the stored energy falls below $E_{R}^{fail}$, a power outage is incurred. On the other hand, if the energy storage device is full, i.e., the amount of stored energy is $E_{R}^{max}$, then the excess of harvested energy is wasted, as it cannot be stored. This situation is called a saturation of the energy storage device. To avoid saturation, the risk of saturation interval $\left[E_{ENI}^{up},E_{R}^{max}\right]$ is defined. It allows the EBC to avoid the waste of energy by overflow of the storage device, serving as a buffer when an increase of the harvested energy occurs. Moreover, the energy stock $E_{S}=E_{ENI}^{down}-E_{R}^{fail}$ is defined as the amount of energy required to ensure the operating of the device during periods without intake energy from the harvester and depends on the application and the energy source characteristics. Therefore, $E_{S}$ is the amount of energy that should be stored in the energy storage device to avoid power outage in the case of energy scarcity periods. The aim of the EBC is therefore to keep the state of charge of the energy storage device in the ENI $\left[E_{ENI}^{down},E_{ENI}^{up}\right]$ when environmental energy is available, thus avoiding the waste of energy by saturation of the storage device, while storing enough energy to survive periods during which no energy is harvested.

We call “quality of service” the application requirements regarding the sensing period, bit error rate, etc. Ensuring the minimum quality of service required by the application necessitates a minimum energy budget per slot denoted by $E_{B}^{min}$. At each execution of the EBC, the energy budget of the next slot *k* is computed as follows:

(2) $$e_{B}\left[k\right]=max\left\{E_{B}^{min},e_{B}\left[k-1\right]+\delta e_{B}\left[k\right]\right\}.$$

where $\delta e_{B}\left[k\right]$ is the energy budget correction, which is calculated according to the current values of $e_{R}$ and $\Delta e_{R}$. The objective of the EM is two-fold: (i) to find an energy budget that achieves an adequate compromise between the discharging rate and quality of service when little environmental energy is available; and (ii) when environmental energy is available, to avoid saturation or to find a good compromise between the charging rate and quality of service. This is done by adjusting the value of the energy budget by an amount that depends on both the residual energy and its variation. The rule base shown in Figure 2b presents the EBC strategy. In this table, $\Delta e_{B}$ is a positive parameter of the EM and corresponds to the energy budget correction when the amount of stored energy is either in the ENI interval or in the risk of saturation interval. As most applications do not perform well under strong variations of the allocated energy budget, choosing $\Delta e_{B}$ requires a compromise between the reactiveness of the EBC and the variability of the allocated energy budget. Indeed, choosing a high value of $\Delta e_{B}$ leads to high reactiveness of the EM, but at the cost of strong variations of the energy budget, which may not be suitable for many applications. On the other hand, choosing low values of $\Delta e_{B}$ leads to the low reactiveness of the EM and smooth energy budget variations. Therefore, the choice of $\Delta e_{B}$ depends on both the energy source and application requirements. Four scenarios can be considered from Figure 2 and are detailed thereafter.

Risk of saturation (R7-8-9):

A risk of saturation occurs when $e_{R}>E_{ENI}^{up}$. To avoid the waste of energy by the overflow of the energy storage device, the EBC increases the energy budget until the residual energy decreases to bring it to a value belonging to the ENI.

Energy neutral interval (R4-5-6):

If the amount of residual energy belongs to the ENI, the EBC goal is to keep the node in the ENO-MAX state. The ENO-MAX state is achieved when the residual energy is kept constant with regard to time, and the EBC thus corrects the energy budget regarding the sign of $\Delta e_{R}$ to keep the node in the ENO-MAX state.

Charging state (R3):

The node is considered to be in the charging state when $e_{R}<E_{ENI}^{down}$ and $\Delta e_{R}$ is positive. The node is thus re-filling its energy stock. In these conditions, the goal of the EBC is to keep the residual energy increasing for the amount of stored energy to be greater than or equal to $E_{S}$, i.e., for the residual energy to reach the ENI in a reasonable time, while allocating a high enough energy budget to ensure a good quality of service. A trade-off must be made between the charging time and the quality of service. Indeed, at one extreme, a conservative policy is to allocate the minimal energy budget while the energy storage device is not fully charged, leading to a quick refill of the storage device at the cost of a low quality of service during the charging phase. On the other hand, allocating almost all the harvested energy will lead to a slow charging rate, but to a good quality of service, regarding the currently-available environmental energy, during the charging phase. As the choice of an appropriate strategy is dependent on the application, a tunable strategy is proposed. The energy budget correction $\delta e_{B}$ is set to a value proportional to the residual energy variation $\Delta e_{R}$, and the proportionality factor is a function of $e_{R}$ denoted by $\mu_{C}\left(e_{R}\right)$ [30]:

(3) $$\mu_{C}\left(e_{R}\right)=M_{C}\left(1-{\left(1-\frac{e_{R}-E_{R}^{fail}}{E_{ENI}^{down}-E_{R}^{fail}}\right)}^{K_{C}}\right).$$

where $M_{C}$ and $K_{C}$ are positive parameters allowing the tuning of the charging strategy. $\mu_{C}$ increases with $e_{R}$, as the more energy is stored, the less conservative we need to be. While $M_{C}$ sets the maximum value of the proportionality factor, $K_{C}$ sets the growth rate of $\mu_{C}$. If $K_{C}=1$, $\delta e_{B}$ increases linearly with $e_{R}$. For values of $K_{C}$ lower than one, the growth rate increases when the residual energy increases, while for values of $K_{C}$ higher than one, the growth rate decreases when $e_{R}$ increases.

Discharging state (R1-2):

The node is considered to be in the discharging state when $e_{R}<E_{ENI}^{down}$ and $\Delta e_{R}$ is negative. The node is thus using its energy stock. In this scenario, a trade-off must be made between the allocated energy budget and the lifetime of the node, i.e., the time it can last before running into a power outage. A conservative policy is to set the energy budget to the minimum required, hence maximizing the lifetime at the cost of a low quality of service. On the other hand, setting the energy budget to an arbitrary high value leads to high quality of service at the cost of short lifetime. Similarly to what has been done for the charging state, a customizable energy management, which can be tuned according to the need of an application, is proposed. The energy budget correction $\delta e_{B}$ is set at a value proportional to the residual energy variation $\Delta e_{R}$, and the proportionality factor is a function of $e_{R}$ denoted by $\mu_{D}\left(e_{R}\right)$ [30]:

(4) $$\mu_{D}\left(e_{R}\right)=M_{D}{\left(1-\frac{e_{R}-E_{ENI}^{down}}{E_{ENI}^{down}-E_{R}^{fail}}\right)}^{K_{D}}.$$

where $M_{D}$ and $K_{D}$ are positive parameters allowing the tuning of the discharging strategy. $\mu_{D}$ decreases with $e_{R}$, as the less energy is stored, the more conservative we must be, and the impacts of $K_{D}$ and $M_{D}$ on the discharging strategy are similar to the ones of $K_{C}$ and $M_{C}$ on the charging strategy.

### 3.2. TC Design

The TC aims to compute the throughput of the node over a time slot to consume the amount of energy specified by the EBC. As wireless communications are usually the most consuming task over all the other tasks such as sensing and computing [6], the throughput of the node given an energy budget is strongly tied to the MAC protocol.

To transmit a single packet, a given MAC protocol typically requires many steps, such as receiving/sending a beacon frame, sending a data frame, receiving an Acknowledgment (ACK) frame, etc. The number of states in which the node can be when communicating using a given protocol is denoted by $N_{S}$. Each state is defined by the combination of the different components’ states (MCU, radio chip, sensors). The time spent in the state $i\in \{1,\ldots ,N_{S}\}$ during a single packet transmission is denoted by $\tau_{i}$, and the corresponding power consumption of the node is denoted by $P_{i}$. The energy cost of the whole process of performing a measurement and sending a single packet is therefore:

(5) $$e_{T}=\sum_{i=1}^{N_{S}}\tau_{i}P_{i}.$$

and the energy consumed by the node over one time slot *k* is:

(6) $$e_{C}\left[k\right]=\frac{T}{T_{WI}\left[k\right]}e_{T}+\left(T-\frac{T}{T_{WI}\left[k\right]}\tau_{T}\right)P_{S}.$$

where $P_{S}$ is the power consumption of the node when all the components are in the sleep state and $\tau_{T}$ is the total time required to perform a measurement and send a packet and is equal to $\tau_{T}=\sum_{i=1}^{N_{S}}\tau_{i}$. Therefore, in order for the consumed energy $e_{C}\left[k\right]$ to be equal to the energy budget $e_{B}\left[k\right]$, the wake-up interval is set to the following value:

(7) $$T_{WI}\left[k\right]=\frac{e_{T}-\tau_{T}P_{S}}{\frac{e_{B}\left[k\right]}{T}-P_{S}}.$$

This equation is obtained by replacing $e_{C}\left[k\right]$ by $e_{B}\left[k\right]$ in Equation (6). The associated throughput, in packets per minute, is thus:

(8) $$THR\left[k\right]=\frac{60}{T_{WI}\left[k\right]}.$$

If we assume a low data-rate application, typical in EH-WSNs, usually MAC protocols are based on pseudo-asynchronous approaches, which makes the estimation of the $\tau_{i}$ values challenging. Indeed, rendezvous schemes incur high variability of the time spent in the idle state and receive state for different packet transmissions. As a consequence of an inaccurate estimation of these values, the energy consumed by the node can be significantly different from the energy budget calculated by the EBC, which can lead to power failures or energy waste. Therefore, in Section 4, a new protocol reducing the energy consumption variability of packet transmission is proposed.

### 3.3. Energy Utilization Coefficient

To evaluate the energy efficiency of different MAC protocols, the Energy Utilization Coefficient (EUC), denoted by ξ, is defined as the ratio of the throughput to the energy budget:

(9) $$\xi \left(e_{B}\right)=\frac{THR}{e_{B}}.$$

It is expressed in packets per minute and per Joule. For notational simplicity, the slot number indication “[k]” is omitted in the rest of this section, and all the following equations refer to a single time slot. The EUC quantifies the achieved throughput of a MAC protocol regarding the available energy budget and is similar to other energy efficiency metrics, e.g., [41,42].

By combining Equations (7)–(9), we obtain:

(10) $$\xi \left(e_{B}\right)=\frac{1}{HT}-\frac{P_{S}}{H}\frac{1}{e_{B}},$$

where *H* (in Joules) is defined by:

(11) $$H=e_{T}-\tau_{T}P_{S}.$$

*H* is a constant particular to a given hardware and MAC protocol. Indeed, the $\tau_{i}$ values depend on the MAC protocol, while the $P_{i}$ values depend on the hardware. Two remarks can be made regarding Equation (10). First, the EUC is not constant for a given hardware and MAC, but increases with the energy budget $e_{B}$. Secondly, the EUC is bounded, as:

(12) $$\xi_{\infty }=\lim_{e_{B}\to \infty }\xi \left(e_{B}\right)=\frac{1}{HT}.$$

From Equation (12), it can be observed that the maximum EUC $\xi_{\infty }$ is higher for small values of *H*. Therefore, the smaller *H* is, the better it is. For the rest of this work, the $P_{i}$ values are assumed to be fixed, and the power consumption in sleep state $P_{S}$ is supposed to be much smaller than the power consumption of the other states $P_{i}$. This assumption holds true for all the WSN platforms. Hence, minimizing *H* is done by minimizing the $\tau_{i}$ values. In order for *H* to be minimal, only the data frame should be sent at each packet transmission. However, most of the MAC protocols introduce an overhead to synchronize the nodes (e.g., the rendezvous process in pseudo-asynchronous MAC protocols) or for error control (e.g., ACK frames). As we will see in the next section, using ULP WuRx allows the minimization of *H* and hence the maximization of the EUC.

## 4. MAC Protocol Leveraging Wake-up Receivers

This section introduces SNW-MAC [43], a protocol for data-gathering star networks that uses ULP WuRx. Traditional protocols use the duty-cycling approach to reduce energy consumption; however, this scheme does not eliminate the energy waste incurred by idle listening and overhearing. Moreover, these protocols are subject to collisions, which reduce their scalability and increase their energy consumption. SNW-MAC leverages ULP WuRx to enable asynchronous communication, minimizing the energy required to transmit a packet and making collisions impossible between packets sent by nodes belonging to the same SNW-MAC-based network. It is assumed that a Physical layer (PHY) providing an error detection mechanism is used. For example, the widespread IEEE 802.15.4 PHY provides a Cyclic Redundancy Check (CRC) error-detecting code.

### 4.1. Design of SNW-MAC

SNW-MAC is an asynchronous scheme that uses the receiver-initiated approach to minimize the energy consumption of WSN nodes. As the power consumption of ULP WuRx has to be orders of magnitude less than the main radio, these devices are usually characterized by low sensitivity and a low data rate [13,44]. For this reason, sending WuBs to a ULP WuRx can be costly energy-wise as it is done at a low bitrate and high transmission power to achieve the same range as the main radio.

**Figure 3:**
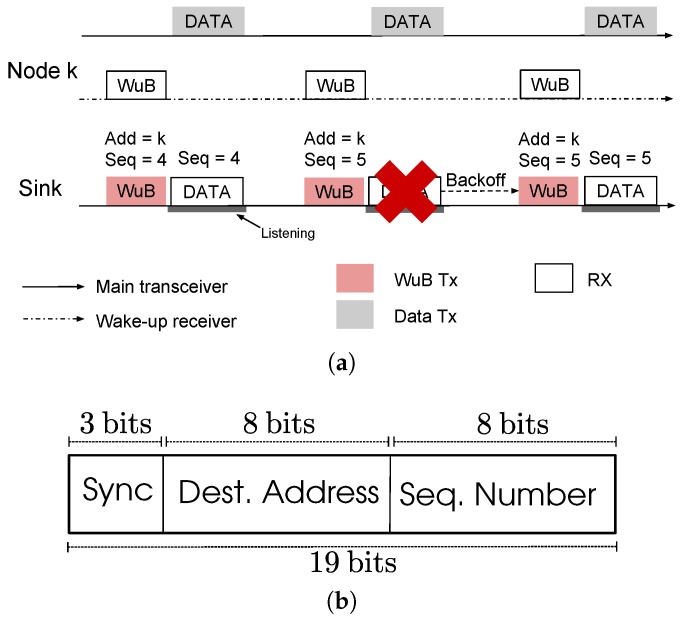
SNW-MAC: packet transmission illustration and Wake-up Beacon (WuB) format. (**a**) Packet transmission using the SNW-MAC. (**b**) WuB format of SNW-MAC.

Packet transmission using SNW-MAC is illustrated by Figure 3a. The sink initializes a communication by sending a WuB containing the address of a specific sensor node and then listens to the channel to receive the data packet. The targeted sensor node is awoken by its ULP WuRx, and starts sending the data packet. Each sensor node piggybacks its wake-up interval in data packets. The sink keeps an updated table that associates for each node its wake-up interval and polls each node at the right time. The sink sets for each node a timer for the wake-up interval piggybacked in data packets and polls the node every time the timer expires. Sensing operations are performed by each node at anytime between two sink polls, ensuring that data are ready to be sent when the sink sends the wake-up beacon. Compared to traditional receiver-initiated protocols, this approach reduces the energy consumption of the sink and the nodes as no rendezvous process is required. The sink energy consumption is further reduced as useless periodic WuBs sending is avoided. Because the wake-up interval is typically a 16-bit integer, minimal overhead is incurred by the piggybacking of this information. Moreover, the sink can use it to monitor the sensor node activity.

WuB format:

The WuB format of SNW-MAC is shown in Figure 3b. A WuB is 19 bits long and is composed of three synchronization bits, the 8-bit address of the node to wake up and an 8-bits sequence number of the expected data packet, used for error control, as explained hereafter.

Error control and retransmission:

By coordinating data packet transmission at the sink, SNW-MAC cancels the risk of collisions compared to traditional pseudo-asynchronous schemes as each node is specifically polled. However, wireless channel interferences may lead to corrupted frames, and energy-efficient error control and packet retransmission is therefore an important issue. As the sink is entirely in charge of coordinating the packet transmission, it is responsible for detecting transmission error and scheduling another attempt. Each WuB embeds an 8-bit sequence number of the expected data packet, as shown in Figure 3b. The sink keeps an updated table that associates for each node the next packet sequence number to poll. When a sensor node ULP WuRx acquires a WuB, it reads both the address and the sequence number. Thanks to the capability of the ULP WuRx to directly recognize the address on the board, it wakes up the node MCU only if the address is valid and then sends to it the sequence number using the serial port. All the packets that have a sequence number lower than the one received are considered as either successfully received or dropped because of a too high number of transmission attempts and are thus erased from the transmission buffer. The data packet that has the sequence number asked by the sink is then sent. The data packet piggybacks its sequence number. When a packet is successfully received by the sink from a given sensor node, the sink checks the data packet sequence number. If the sequence number of the data packet is the one expected by the sink, then the sink increments the sequence number associated with this node. When the sink detects a transmission failure, e.g., the received data packet is corrupted, it does not increment the sequence number and sets a random backoff. When the backoff expires, it initiates a new communication using the same sequence number, as illustrated by Figure 3a. Compared to traditional error-control schemes that require ACK frames, the energy overhead is significantly reduced for sensor nodes as they do not need to listen to ACK frames after each data packet transmission. On the sink side, as no ACK frame is sent, energy is also saved. Nonetheless, this energy saving is counterbalanced by longer WuBs sent by the sink due to the sequence number.

Using SNW-MAC, only the data frame is sent by the nodes, thus minimizing the per-packet energy consumption and the *H* value introduced in Section 3.2. Moreover, the per-packet energy consumption variability is also minimized if the data frame length does not change. Indeed, the only possible cause of energy consumption variability is due to retransmissions. Having a low energy consumption variability is important to allow the EM to accurately control the energy consumption of the node.

### 4.2. Analytical Study of Scalability

In this section, the scalability of SNW-MAC and traditional pseudo-asynchronous MAC protocols is evaluated in the context of star networks. The emphasis is put on the sink, which is in charge of gathering the packets from all the sensors. This section compares the achievable packet reception rates when SNW-MAC is used and when pseudo-asynchronous MAC protocols are used, in order to study how sustainable the proposed approach is with regard to the sink compared to these protocols. The number of nodes that compose the network is denoted by *N* (not including the sink), and the packet generation rate is modeled by a Poisson distribution of parameter λ packets per minute. Next, the expressions of the packet arrival rate at the sink are derived for SNW-MAC and pseudo-asynchronous MAC protocols when it is assumed that the only cause of packet loss is collisions and that all collisions are destructive, i.e., lead to corrupted packets.

SNW-MAC:

Collisions between packets sent by nodes belonging to the same SNW-MAC-based network are impossible as the sink specifically polls each node. Nonetheless, as receiving a packet requires a non-null duration, the receiving rate is still bounded. The total time required to receive a packet is denoted by $\tau_{R}$ and is defined by:

(13) $$\tau_{R}=\tau_{d}+\tau_{o},$$

where $\tau_{d}$ is the time required to receive the data payload and $\tau_{o}$ is the overhead incurred by the hardware and the protocol at each packet reception (WuB sending, radio setup, turn-around time, software overhead). The maximum receiving rate in packets per minute is thus:

(14) $$\Gamma =\left\lfloor \frac{60}{\tau_{R}}\right\rfloor ,$$

where ⌊·⌋ is the floor function. We assume that the packet generation rates of the nodes are independent of each other and are modeled by Poisson distributions of mean λ packets per minute, and we denote by *A* the aggregate rate. As Poisson distributions are stable by sum, *A* follows a Poisson distribution of mean Nλ packets per minute. However, because the maximum receiving rate of the sink is Γ, the receiving rate of the sink, denoted by *R*, is modeled by the following distribution:

(15) $$\mathrm{Pr}\left(R=k\right)=\left\{\begin{array}{cc} \mathrm{Pr}\left(A=k\right)=\frac{{\left(\lambda N\right)}^{k}}{k!}e^{-\lambda N} & 0\leq k<\Gamma \\ \sum_{i=\Gamma }^{\infty }\mathrm{Pr}\left(A=i\right)=\sum_{i=\Gamma }^{\infty }\frac{{\left(\lambda N\right)}^{i}}{i!}e^{-\lambda N} & k=\Gamma \\ 0 & k>\Gamma \end{array}\right.$$

Indeed, the sink saturates when the receiving packet rate reaches Γ, and therefore, higher receiving packet rates are impossible as the sink cannot poll the nodes quickly enough. The average packet rate is thus:

(16) $$\gamma_{SNW-MAC}=E\left[R\right]=\sum_{k=0}^{\infty }k\mathrm{Pr}\left(R=k\right)=\sum_{k=1}^{\Gamma -1}k\frac{{\left(\lambda N\right)}^{k}}{k!}e^{-\lambda N}+\Gamma \sum_{k=\Gamma }^{\infty }\frac{{\left(\lambda N\right)}^{k}}{k!}e^{-\lambda N}.$$

As:

(17) $$\sum_{k=\Gamma }^{\infty }\frac{{\left(\lambda N\right)}^{k}}{k!}=e^{\lambda N}-\sum_{k=0}^{\Gamma -1}\frac{{\left(\lambda N\right)}^{k}}{k!},$$

we finally have:

(18) $$\gamma_{SNW-MAC}=e^{-\lambda N}\sum_{k=0}^{\Gamma -1}k\frac{{\left(\lambda N\right)}^{k}}{k!}+\left(1-e^{-\lambda N}\sum_{k=0}^{\Gamma -1}\frac{{\left(\lambda N\right)}^{k}}{k!}\right)\Gamma .$$

Pseudo-asynchronous MAC:

Using traditional pseudo-asynchronous MAC, the sink periodically wakes up to receive data packets, and the sink wake-up interval is denoted by $T_{S}$. The time is thus divided into equal length time slots of duration $T_{S}$. When a node generates a packet, it typically tries to send it at the sink’s next wake-up. The number of packets, denoted by *X*, generated by a given node over a time slot can be modeled by a Poisson distribution of parameter $\frac{\lambda T_{S}}{60}$. Therefore, the probability that a node generates packets in a time slot is:

(19) $$p_{g}=\mathrm{Pr}\left(X\geq 1\right)=1-\mathrm{Pr}\left(X=0\right)=1-e^{-\frac{\lambda T_{S}}{60}}.$$

Let *Y* be the number of nodes that have generated packets over a time slot. *Y* can be modeled by a binomial distribution of parameter $p_{g}$. As the *Y* nodes will try to send a packet at the next sink wake-up and if we assume that all the collisions are destructive, the number of packets received by the sink during a time slot is a function of *Y* denoted by $R^{\prime }\left(Y\right)$ and defined by:

(20) $$R^{\prime }\left(Y\right)=\left\{\begin{array}{cc} 0 & \;\mathrm{if}\;Y=0\\ E\left[X|X\geq 1\right] & \;\mathrm{if}\;Y=1\\ 0 & \;\mathrm{if}\;Y>1 \end{array}\right.$$

the last case being the collision scenario. Furthermore:

(21) $$E\left[X|X\geq 1\right]=\sum_{k=1}^{\infty }k\mathrm{Pr}\left(X=k|X\geq 1\right),$$

and for k≥1:

(22) $$\mathrm{Pr}\left(X=k|X\geq 1\right)=\frac{\mathrm{Pr}\left(X=k,X\geq 1\right)}{\mathrm{Pr}\left(X\geq 1\right)}=\frac{\mathrm{Pr}\left(X=k\right)}{p_{g}}$$

leading to:

(23) $$E\left[X|X\geq 1\right]=\frac{E\left(X\right)}{p_{g}}=\frac{\lambda T_{S}}{60p_{g}}.$$

Therefore, the average number of packets received during a time slot is:

(24) $$E\left[R^{\prime }\left(Y\right)\right]=\sum_{i=1}^{n}R^{\prime }\left(Y=i\right)\mathrm{Pr}\left(Y=i\right)=E\left[X|X\geq 1\right]\mathrm{Pr}\left(Y=1\right)=\frac{N\lambda T_{S}}{60}e^{-\left(N-1\right)\frac{\lambda T_{S}}{60}},$$

and the average receiving rate $\gamma_{PAM}$ in packets per minute is thus:

(25) $$\gamma_{PAM}=\frac{60E\left[R^{\prime }\left(Y\right)\right]}{T_{S}}=N\lambda e^{-\left(N-1\right)\frac{\lambda T_{S}}{60}}.$$

**Figure 4:**
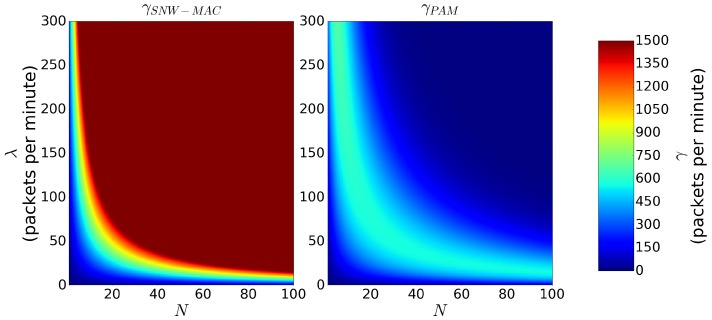
$\gamma_{SNW-MAC}$ and $\gamma_{PAM}$ as a function of *N* and λ when $T_{S}=\tau_{R}=40\;$ms.

Figure 4 shows $\gamma_{SNW-MAC}$ and $\gamma_{PAM}$ for values of *N* ranging from 0–100 and values of λ ranging from values of 0–300 packets per minute. In real scenarios, the wake-up interval of pseudo-asynchronous protocols $T_{S}$ is usually set to a much higher value than $\tau_{R}$ to save energy. However, in order to compare SNW-MAC to the best case scenario of pseudo-asynchronous protocols regarding scalability, both $\tau_{R}$ and $T_{S}$ were set to 40ms, leading to Γ=1500 packets per minute for SNW-MAC. As we can see, $\gamma_{SNW-MAC}$ increases until reaching Γ. The sink then saturates, and the receiving packet rate stops increasing. On the other hand, $\gamma_{PAM}$ first increases with λ and *N*, but decreases after reaching a maximum because of collisions, limiting its scalability. Moreover, the maximum reached by $\gamma_{PAM}$ is 674packets per minutes, which is more than twice smaller than the maximum reached by $\gamma_{SNW-MAC}$. These numerical results show the better scalability of SNW-MAC, even when compared to the best case scenario of pseudo-asynchronous MAC protocols. In the case where $T_{S}$ is set to 250ms and $\tau_{R}$ to 40ms, which are typical real scenario values, numerical results show that the highest packet rate achieved by pseudo-asynchronous MAC protocols is 300packets per minute, which is 5-times lower than SNW-MAC.

## 5. Experimental Setup

The experimental setup used to evaluate our approach is presented in this section. First, the architecture of the nodes that compose the testbed is presented. Then, details on the ULP WuRx implementation are given. Finally, the designs of two state-of-the-art MAC protocols, to which SNW-MAC is compared, are presented.

### 5.1. Node Architecture

**Figure 5:**
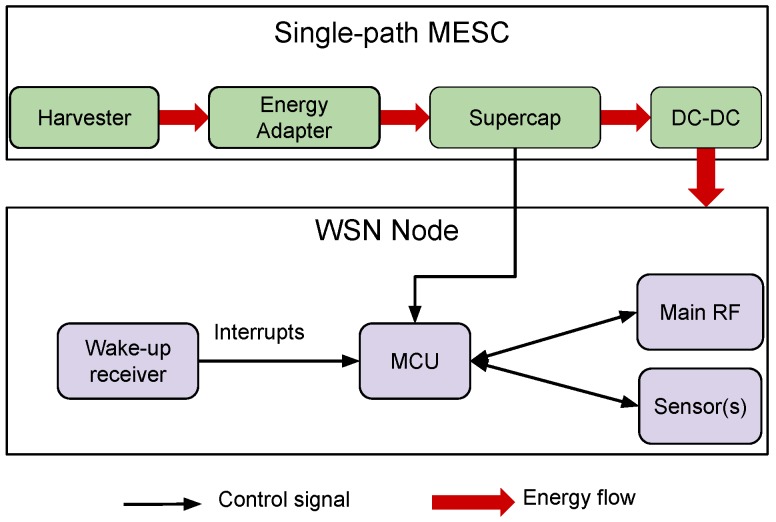
Hardware architecture of a WSN node using the Multiple Energy Source Converter (MESC) architecture and a WuRx. UPMA, Unified Radio Power Management Architecture.

**Figure 6:**
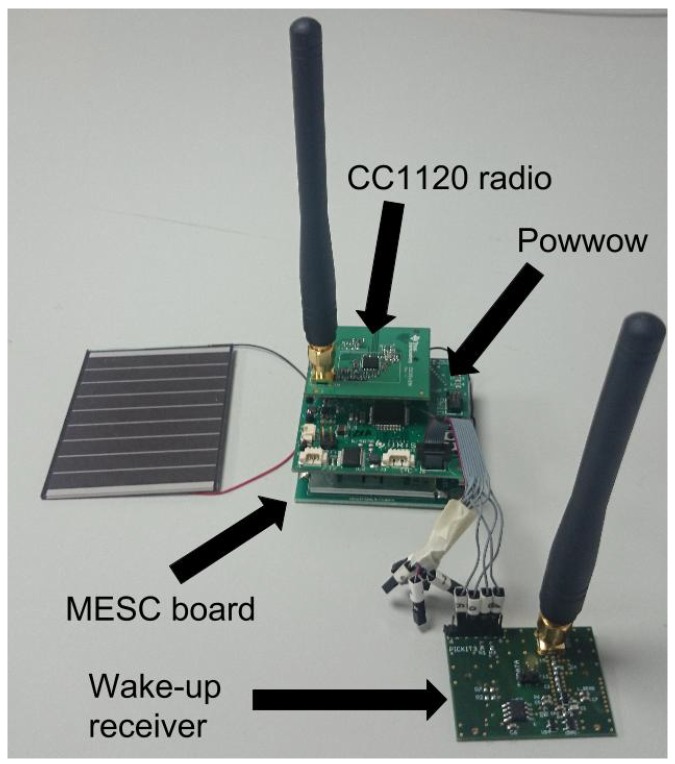
PowWow node equipped with an Ultra Low Power (ULP) WuRx.

Multiple EH-WSN platforms have been proposed by academia and industry over the last decade. In this work, we consider a single-path architecture version of the Multiple Energy Source Converter (MESC) architecture proposed in [45]. In the single-path architecture, there is only one energy storage device, and all the harvested energy is used to charge the storage device, which directly powers the node through a DC-DC converter. Figure 5 shows the block architecture of MESC that can be used with a variety of energy harvesters (e.g., photovoltaic cells, thermoelectric generators and wind turbines) using the appropriate energy adapter to normalize the output energy. Supercapacitors were chosen as storage devices as they are more durable and offer a higher power density than batteries [46]. In this work, the PowWow platform [47], based on the MESC architecture and equipped with a Texas Instruments CC1120 radio chip, is used as the testbed. The energy storage device is a 0.9F supercapacitor with a maximum voltage of 5.0V, and the minimum voltage required to power the node is 2.8V. PowWow embeds a voltage measurement chip, the INA3221 from Texas Instruments, which allows measurement of the supercapacitor voltage, denoted by $V_{C}$, with a precision of 0.1mV. The residual energy $e_{R}$ can thus easily be computed as follows:

(26) $$e_{R}=\frac{1}{2}C{V_{C}}^{2},$$

where *C* is the supercapacitor capacitance. When SNW-MAC is evaluated, the ULP WuRx is added to the node, and the so-obtained mote is shown in Figure 6. The EM introduced in Section 3 was implemented on PowWow, in addition to SNW-MAC introduced in Section 4 and two others state-of-the-art MAC protocols presented in Section 5.3. The parameters used for experimentations are shown in Table 1.

**Figure 7:**
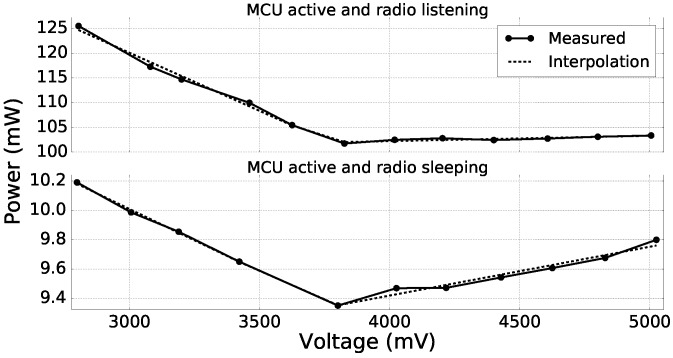
Power consumption of the node for different input voltages of the DC-DC converter when (i) the MCU is active and the radio is listening and when (ii) the MCU is active and the radio is sleeping.

As the supercapacitor supplies the node via a DC-DC converter as shown in Figure 5 and the efficiency of the DC-DC converter varies with the input voltage, the power consumed by the node depends on the charge of the supercapacitor. Therefore, the power consumption of each of the $N_{S}$ states (introduced in Section 3.2) was measured for different input voltages of the DC-DC converter ranging from 2.8V–5.0V, and piecewise linear interpolation was used to get the $P_{i}$ values as functions of the supercapacitor voltage. Figure 7 shows the so-obtained measures and the corresponding interpolated functions for two states of the node. As we can see, piecewise linear interpolation permits an accurate modeling of the node power consumption.

### 5.2. Ultra-Low Power Wake-Up Receiver

Each wireless sensor node is equipped with a ULP WuRx presented in [13] only when the proposed approach is evaluated. This ULP WuRx employs On-Off Keying (OOK) modulation, the simplest form of Amplitude-Shift Keying (ASK) modulation, in which digital data are represented by the presence or absence of the carrier wave. The analog front-end of the receiver is designed for the 868-MHz frequency band and has a sensitivity measured to be −55dBm at a bitrate of 1kbps. The computational capabilities of the ULP WuRx are achieved by the use of a ULP 8-bit microcontroller, the PIC12LF1552 from Microchip, which was selected for its low current consumption (20nA in sleep mode), fast wake-up time (approximately 130μs at 8MHz) and for the serial port supporting I2C and SPI, allowing easy communications with the node MCU and enough computational capability for parsing data and commands. When a carrier is detected, the front-end wakes up the microcontroller that reads the address embedded in the WuB and performs address matching. If the received address is not valid, the microcontroller goes back to a sleep state. If it is the valid one, it wakes up the node MCU using an interrupt.

### 5.3. State-Of-The-Art MAC Protocols Used for Comparison

**Figure 8:**
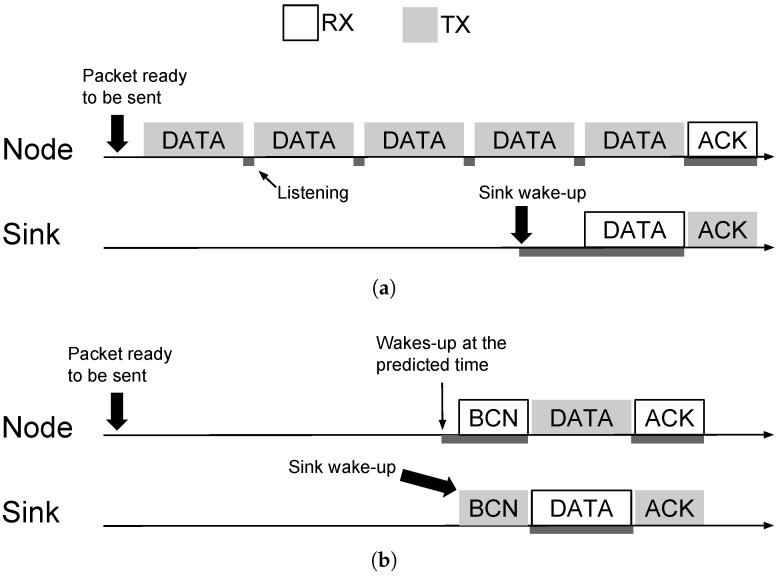
UPMA-X-MAC and PW-MAC protocols. BCN, Beacon. (**a**) Packet transmission using UPMA-X-MAC. (**b**) Packet transmission using PW-MAC.

The SNW-MAC protocol described in the previous section is compared to PW-MAC [9] and the Unified Radio Power Management Architecture (UPMA)-X-MAC [48], two well-known state-of-the-art pseudo-asynchronous MAC protocols. Figure 8a illustrates a packet transmission using the transmitter-initiated UPMA-X-MAC from UPMA. The node initiates the communication by continuously sending the data packet until an ACK frame from the sink is received. On its side, the sink periodically wakes up and listens to the channel. If it detects activity, it waits until the incoming data packet is fully received and then sends the ACK frame.

PW-MAC is a receiver-initiated protocol that focuses on energy efficiency at both the receiver and transmitter sides. A simplified version of the protocol is used in this study as only downlink transmissions are considered. The packet transmission using PW-MAC is shown in Figure 8b. At the receiver side, the sink periodically wakes up and sends a Beacon (BCN) frame. At the transmitter side, each node accurately predicts the time at which the sink will wake up. If a packet needs to be sent, the node wakes up just before the next beacon is sent by the sink. Once the beacon is acquired, the node sends the data packet and waits for the ACK frame. At each packet transmission, a prediction error is computed, and the node updates its prediction time according to this error.

## 6. Experimental Results

This section starts by analyzing the WuRx power consumption. Then, results of the microbenchmarks performed to provide detailed insights into the energy cost of the transmission and reception of a packet using the evaluated protocols are exposed, and these results are used to compute the *H* and $\xi_{\infty }$ values related to the EUC metric defined in Section 3. The energy consumption of the sink incurred by the evaluated protocols is then studied. Next, the benefits of SNW-MAC are shown by comparing it to the two state-of-the-art MAC protocols introduced in the previous section. Finally, our scheme is evaluated under variable energy harvesting conditions to show the benefits of the EM in collaboration with the MAC protocols and the higher performance of the proposed approach.

### 6.1. Energy Consumption of the Wake-Up Receiver

**Figure 9:**
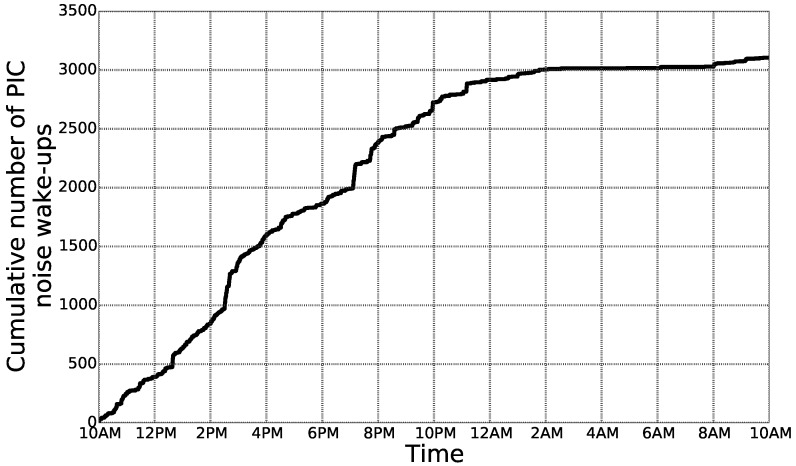
False wake-ups of the PIC over a 24-h period in an indoor environment.

One of the requirements of a ULP WuRx is very low power consumption as it is always active, even when all the other components are in the sleep state. The power consumption of the ULP WuRx was measured to be 1.83μW when the radio front-end was active and the PIC was in sleep state and 284μW when the PIC was active at 3.3V and was parsing the received data at 2MHz. Therefore, the ULP WuRx power consumption becomes significant when the PIC is active. At each wake-up, the PIC is active for 19ms to perform address matching. Hence, the energy consumed by the ULP WuRx at each wake-up of the PIC is 5.40μJ. If we consider a typical node, not using a ULP WuRx, but using the duty-cycling approach with a duty-cycle set to a typical value of 0.05% and consuming 100mW when the transceiver is active, then the total energy consumed by this node over a period of 24h is 4.32J. This amount of energy corresponds to more than $8\times 10^{5}$ wake-ups of the PIC. The number of “false” PIC wake-ups, i.e., wake-ups not caused by WuBs, but by the wireless channel noise, was measured over a period of 24h in an indoor environment. Figure 9 shows the total cumulative number of false wake-ups according to time. It is not surprising to observe that most of the false wake-ups happen during the daytime. In total, 3110 false wake-ups were counted over a 24-h period, which is two orders of magnitude below the previously-considered scenario assuming a typical 0.05% duty-cycle. Moreover, no false wake-ups of the main MCU happened. These results show the importance of the microcontroller embedded in the ULP WuRx. Indeed, performing address matching by a ULP microcontroller avoids numerous false wake-ups of the node MCU, whose power consumption is significantly higher.

### 6.2. Energy Microbenchmarks

To evaluate the energy efficiency of a MAC protocol, it is important to measure the energy consumption of the transmission and the reception of a single packet. Therefore, the energy traces of both operations were measured for the three evaluated protocols, by capturing the voltage drop across a 10.2Ω resistor in series with a 3.5-V power supply using an Agilent Technologies MSO-X-3024A oscilloscope. In addition to allowing detailed analysis of the energy consumption, these microbenchmarks were used to set the $\tau_{i}$ values introduced in Section 3.2 and to compute the *H* and $\xi_{\infty }$ values related to the EUC metric.

**Figure 10:**
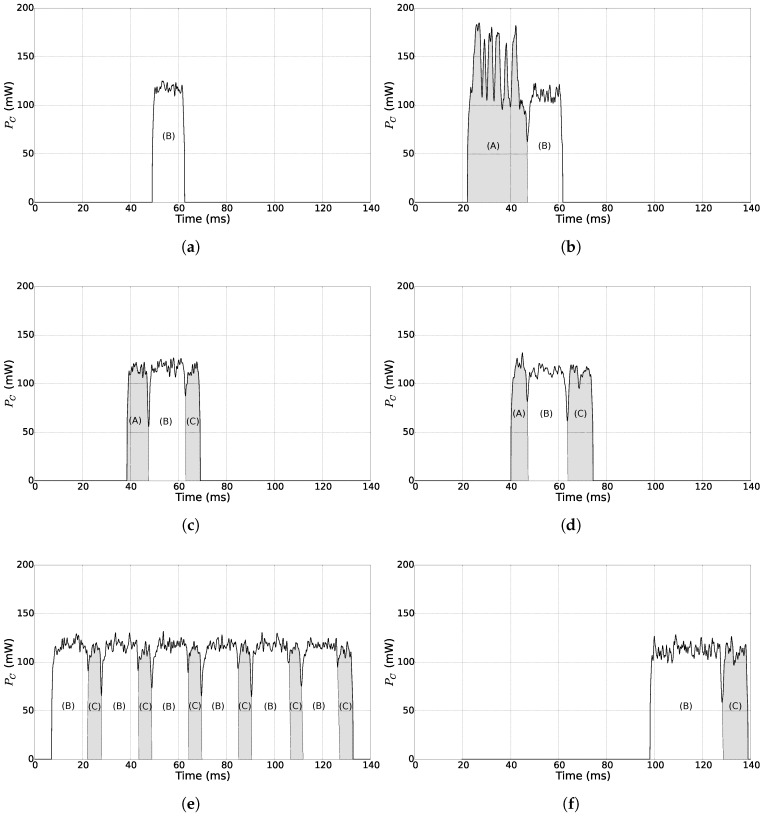
Microbenchmarks of the MAC protocols. (A)–(C) respectively correspond to the transmission/reception of a beacon/WuB, data frame and ACK frame. (**a**) SNW-MAC: transmission of a data packet. (**b**) SNW-MAC: reception of a data packet. (**c**) PW-MAC: transmission of a data packet. (**d**) PW-MAC: reception of a data packet. (**e**) UPMA-X-MAC: transmission of a data packet. (**f**) UPMA-X-MAC: reception of a data packet.

The results of the measurements are exposed by Figure 10, in which $P_{C}$ is the power consumption of the node. Figure 10a shows that sending a data packet using the proposed SNW-MAC protocol achieves the lowest power consumption compared with the other protocols, as it requires only the sending of the data frame (B). The reception of the WuB does not appear in this figure, as the power consumption of the WuRx when decoding a WuB is 284μW, which is too low to be visible on the scale of Figure 10a. Moreover, the energy cost of sending a packet is constant if the data payload length is fixed. Regarding the sink in Figure 10b, the two stages of a packet reception, sending the WuB (A) then receiving the data frame (B), can be seen in this figure. As sending the WuB is done at a lower bitrate and higher transmission power than for non-WuB frames, polling a node is energetically expensive for the sink. This result motivates the piggybacking of the wake-up interval of each node in data packets, allowing the sink to poll them only at the right time (see Section 4).

Figure 10c,d shows respectively the energy cost of a packet transmission and reception using PW-MAC. Sending a packet with this protocol requires the receiving of a beacon (A) and an ACK (C) frame, making the energy cost of sending a packet higher than with SNW-MAC. Moreover, the sender wakes up a short time before the sink transmits a beacon to prevent prediction errors. This time interval varies at each transmission, leading to a non-constant energy cost per packet transmission. The prediction error becomes significant due to the clock drift, and when it exceeds a fixed threshold, an update of the prediction state is triggered, leading to even higher energy consumption. Regarding the sink, receiving a data packet does not require the sending of a WuB and is thus less energetically expensive than with SNW-MAC. Nonetheless, SNW-MAC does not require the transmission of an ACK frame, which partially counterbalances the energy overhead incurred by the WuB transmission when compared to PW-MAC.

Figure 10e shows the energy cost of sending a packet for a node using UPMA-X-MAC. In this case, the packet was successfully received by the sink at the sixth attempt. For each attempt, the two stages, sending the data packet (B) and listening for an ACK (C), can be seen. As shown in Figure 10f, the sink woke up during the fifth attempt (B) and thus did not receive the complete data packet. It stayed awake to receive it at the next attempt (B) and sent an ACK (C). The cost of sending one packet with UPMA-X-MAC greatly varies for different transmissions because of the randomness of the sink wake-up time relative to the node transmission starting time. On average, a node has to wait half of the wake-up interval of the sink before a data packet is successfully received. This makes the sending of a packet with this protocol energetically more expensive than with SNW-MAC or PW-MAC. On the sink side, the energy cost of a packet reception is also highly variable and requires on average the listening of one and a half data packets in addition to the transmission of an ACK frame.

Using these microbenchmarks, the $\tau_{i}$ values introduced in Section 3.2 were measured, and *H* and $\xi_{\infty }$ were calculated for the different MAC protocols using the lowest measured values of the $P_{i}$, leading to the best achievable values of *H* and $\xi_{\infty }$. Table 2 presents the obtained results. It can be observed that using SNW-MAC allows a significantly better use of the energy budget. Indeed, SNW-MAC permits values of *H* (respectively $\xi_{\infty }$) more than twice smaller (respectively bigger) than with PW-MAC and more than nine-times smaller (respectively bigger) than with UPMA-X-MAC.

Energy overhead of the EM:

The EM is periodically executed by each node and therefore incurs an energy overhead. Using micro-benchmarks, it was measured that each execution of the EM consumes 207.41μJ at most. For the rest of this work, the duration between two executions of the EM *T* is set to 120s, and the power consumption overhead incurred by the EM is thus equivalent to a constant power draw of 1.73μW, which is similar to the power consumption of state-of-the-art electronic components for WSN nodes in the sleep state.

### 6.3. Energy Consumption of the Sink

To evaluate the energy consumption of the sink incurred by the evaluated MAC protocols, the energy cost per received packet was evaluated. Using SNW-MAC, the sink only wakes up to poll a sensor node to receive a packet, and therefore, the energy cost per packet received is constant. However, using a pseudo-asynchronous MAC protocol, such as UMPA-X-MAC or PW-MAC, the sink must periodically wake up to check for incoming packets. If the average rate at which the sink receives packets is denoted by γ and if the cost of waking up and receiving a packet is denoted by $e_{RX}$ and the cost of waking up and not receiving any packet is denoted by $e_{W}$, then the average power consumption of the sink incurred by packet reception is:

(27) $${\bar{P}}_{C,sink}=e_{RX}\gamma +e_{W}\left(\frac{1}{T_{S}}-\gamma \right).$$

The average energy cost per received packet is therefore:

(28) $${\bar{e}}_{P}=e_{RX}+e_{W}\left(\frac{1}{\gamma T_{S}}-1\right).$$

**Figure 11:**
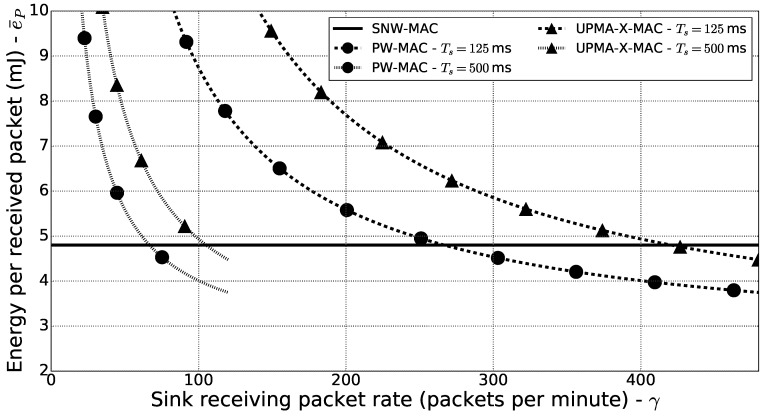
Energy consumption per received packet for the evaluated protocols.

Using the microbenchmarks presented previously, the values of $e_{RX}$ and $e_{W}$ were measured for UPMA-X-MAC and PW-MAC, and the energy cost of receiving a packet using SNW-MAC was also measured. Figure 11 shows the energy cost per received packet for the three evaluated protocols, for different values of γ and for values of $T_{S}$ of 125ms and 500ms. These results were plotted using Equation (28), for values of γ in the range $\left[0,\frac{1}{T_{S}}\right]$. It can be seen that if γ is lower than 66 packets per minute, then SNW-MAC enables lower energy consumption on the sink side, despite the higher power needed to transmit WuB and even when the wake-up interval is set to 500ms. Moreover, when the wake-up interval of pseudo-asynchronous protocols is decreased, which is required if the packet rate is high to reduce collision risk or if low latency is required, the energy cost per packet received significantly increases for UMPA-X-MAC and PW-MAC, as can be seen in Figure 11. In the monitoring applications, which is the focus of this work, low packet rates are expected, and in this case, Figure 11 shows that SNW-MAC enables lower power consumption on the sink side.

### 6.4. Evaluation on a Star Network

**Figure 12:**
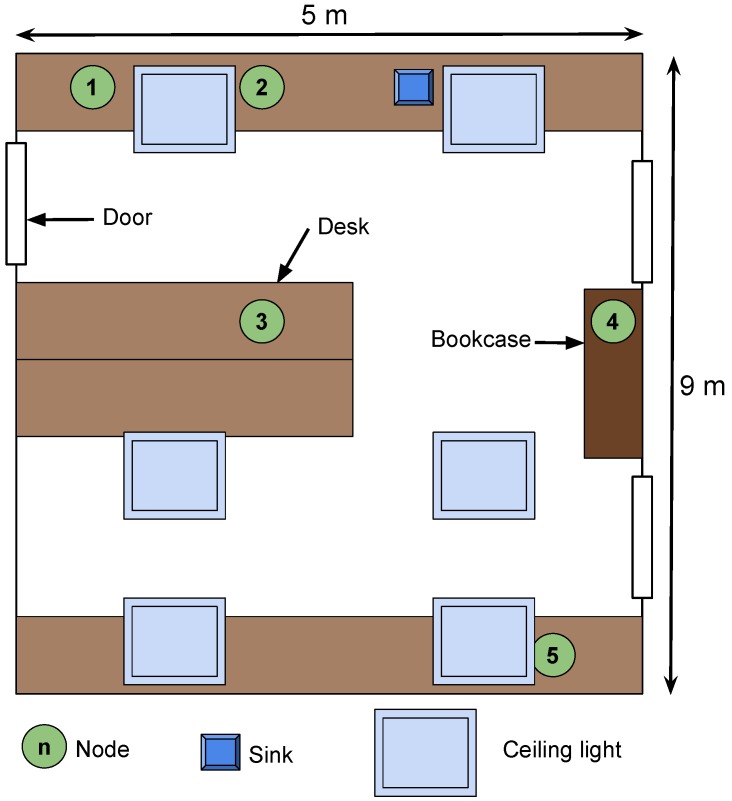
Setup of the star network.

**Figure 13:**
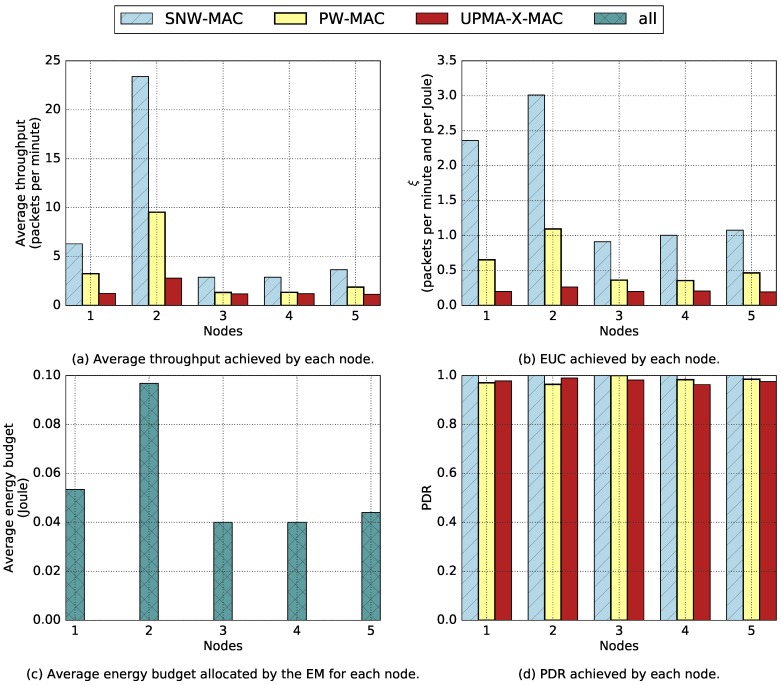
Results of the experimentations on a star network.

The proposed EM and the evaluated MAC protocols were implemented on a testbed made of six PowWow nodes including one sink, in a star topology. The nodes were deployed in a room with no windows and were exclusively powered by indoor fluorescent light, which achieved a luminance of 500lx, allowing reproducibility of the experiments. Each node, except the sink, was equipped with a solar panel Sanyo AM-5913CAR-SCE, of size 60.1mm by 55.1mm. The sink was battery powered. Moreover, the nodes were deployed under different lighting conditions, as shown in Figure 12. Nodes 1, 2 and 5 were located on desks, directly under the ceiling lights, while Node 3 was deployed in a more shadowed area, and Node 4 was located on a bookcase, close to the ceiling, thus receiving less light than the others. Each experiment lasted for 3h and was performed during daytime, and the PowWow nodes have been equipped with a ULP WuRx only when the SNW-MAC protocol was evaluated. Figure 13 shows the obtained results, where Figure 13a presents the throughput, in packets per minute, achieved with the different MAC protocols. SNW-MAC significantly outperforms the two other protocols, allowing up to two-times higher throughput than PW-MAC for Node 2 due to the lower energy cost of packet transmissions. The performance of SNW-MAC is confirmed by Figure 13b, which shows that the EUC is much higher for SNW-MAC, revealing a better use of the energy budget. It is not surprising to notice that the results obtained for each node are strongly linked to the average energy budget allocated by the EBC shown in Figure 13c. As the amount of harvested energy varies for different nodes, the average allocated energy budget also differs. Indeed, Nodes 3 and 4 were placed in shadowed areas and therefore received less environmental energy than the others, leading to lower average energy budgets. Node 2 was on a shelf, closer to the light source, and therefore harvested more energy. Finally, Figure 13d shows the Packet Delivery Ratio (PDR) achieved by the three protocols. A PDR of 100% is achieved for all nodes that use SNW-MAC, while failed transmissions occur for the two other protocols. These results show that the use of ULP WuRx enables the design of highly-reliable protocols.

### 6.5. Evaluation under Variable Light Conditions

**Figure 14:**
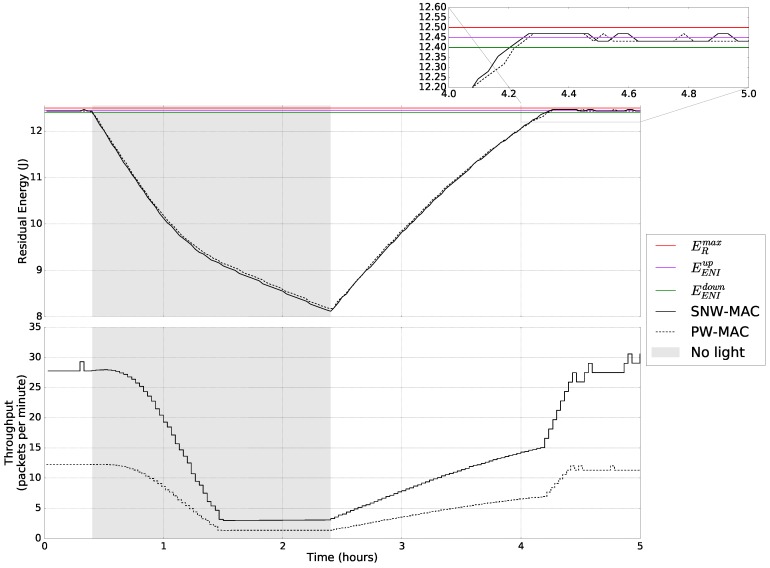
Behavior of the EM and achievable throughput with variable lighting conditions.

The benefits in terms of achievable throughput of the proposed approach have been evaluated under variable light conditions. The residual energy of a single sensor node was tracked when exposed to fluorescent lighting, typical of an indoor environment, then without any available environmental energy (the lights were off) for 2h and finally exposed to indoor light again. The whole experiment lasted for 5h, starting with the storage fully charged, and the results are shown in Figure 14. In this evaluation, only PW-MAC has been compared to SNW-MAC, as it allows higher throughput than UPMA-X-MAC, as we have previously seen.

Figure 14 shows that the EM successfully adapts the throughput of the node to keep it sustainable. In the first part of the experiment, i.e., before the light is turned off, and in the last part, i.e., after 4.3h, the energy buffer is full, and environmental energy is available. In that case, the EM aims at keeping the amount of residual energy in the ENI interval. When the residual energy is in the range $\left[E_{ENI}^{up},E_{R}^{max}\right]$, the energy budget is increased by an amount of $\Delta e_{B}$, in order to avoid energy waste, which corresponds to the rules (R7) and (R8) of Figure 2b. When the residual energy is in the ENI interval, the energy budget is either left unchanged or adjusted by $\pm \Delta e_{B}$ according to the sign of $\Delta e_{R}$. These cases correspond to the risk of saturation and energy neutral interval scenarios introduced in Section 3.1.

When no environmental energy is available, the energy budget is progressively decreased, following the rule R1. One and a half hours after the beginning of the experiment, the minimal energy budget is reached, and the energy budget is no longer decreased to ensure minimum quality of service. However, the energy buffer keeps discharging, as no energy is harvested. This corresponds to the discharging state scenario introduced in Section 3.1. When environmental energy is available, 2.4h after the beginning of the experiment, the energy buffer starts recharging, and the energy budget is progressively increased until the ENI interval is reached, which corresponds to the rule R3 and to the charging state scenario. Once the ENI interval is reached, the energy budget is still increased to avoid saturation, which corresponds to the rules R7 and R8.

From this evaluation, we observe that the throughput using SNW-MAC is in all conditions higher than with PW-MAC, showing the better energy efficiency of SNW-MAC. Especially, the throughput of the proposed approach is up to 2.5-times higher than PW-MAC in periods where harvested energy is available.

These results demonstrate the ability of the EM to achieve energy neutrality with different MAC protocols and the benefits of its combination with the highly efficient SNW-MAC protocol, which exploits a ULP WuRx to enable asynchronous communication.

## 7. Conclusions

In this work, an energy manager combined with an asynchronous MAC protocol has been proposed for energy harvesting wireless sensor networks. The proposed solution leverages two complementary technologies, energy harvesting and ultra-low power wake-up receivers, to increase the energy efficiency of wireless sensor networks and to enable energy neutrality. This new scheme is designed to be implemented on real hardware and therefore solely requires the measure of the residual energy achieving a negligible overhead. The proposed approach has been experimentally validated in the context of data-gathering sensor networks with a star topology and compared with two state-of-the-art MAC protocols. Experimental results show that a 2.5 gain in term of throughput can be achieved by SNW-MAC compared to PW-MAC The energy efficiency was evaluated using a new metric introduced in this work, the energy utilization coefficient. Moreover, the better scalability of the proposed MAC protocol compared to traditional pseudo-asynchronous MAC has been analytically demonstrated.

## Figures and Tables

**Table 1:** Parameters used for the experiments.

Parameters		Values
MAC	Sink wake-up interval (UPMA-X-MAC and PW-MAC)	250ms
	Maximum number of retransmissions	2
PHY	WuB bitrate	1kbps
	Data/ACK/beacon bitrate	20kbps
	WuB transmission power	12.5dBm
	Data/ACK/beacon transmission power	−6dBm
EBC	$M_{C}$	0.01
	$K_{C}$	2.0
	$M_{D}$	0.5
	$K_{D}$	2.0
	$\Delta e_{B}$	5mJ
	$E_{R}^{fail}$	3.528J
	$E_{ENI}^{down}$	12.40J
	$E_{ENI}^{up}$	12.45J
	$E_{R}^{max}$	12.50J
	$E_{B}^{min}$	0.04J

**Table 2:** Best values of *H* and $\xi_{\infty }$ for the different MAC protocols.

Protocol	*H* (Joule)	$\xi_{\infty }$ (Packets Per Minute and Per Joule)
UPMA-X-MAC	0.0125	0.666
PW-MAC	0.00313	2.660
SNW-MAC	0.00135	6.156
